# The deep learning radiomics nomogram helps to evaluate the lymph node status in cervical adenocarcinoma/adenosquamous carcinoma

**DOI:** 10.3389/fonc.2024.1414609

**Published:** 2024-12-13

**Authors:** Mei Ling Xiao, Le Fu, Ting Qian, Yan Wei, Feng Hua Ma, Yong Ai Li, Jie Jun Cheng, Zhao Xia Qian, Guo Fu Zhang, Jin Wei Qiang

**Affiliations:** ^1^ Department of Nuclear Medicine and PET Center, The Second Affiliated Hospital of Zhejiang University School of Medicine, Hangzhou, China; ^2^ Department of Radiology, Jinshan Hospital, Fudan University, Shanghai, China; ^3^ Department of Radiology, Shanghai First Maternity and Infant Hospital, Tongji University School of Medicine, Shanghai, China; ^4^ Department of Radiology, International Peace Maternity and Child Health Hospital, Shanghai Jiaotong University School of Medicine, Shanghai, China; ^5^ College of Information Engineering, Zhejiang University of Technology, Hangzhou, Zhejiang, China; ^6^ Departments of Radiology, Obstetrics and Gynecology Hospital, Fudan University, Shanghai, China

**Keywords:** magnetic resonance imaging, cervical adenosquamous carcinoma, cervical adenocarcinoma, radiomics, deep learning, lymph node metastasis

## Abstract

**Objectives:**

The accurate assessment of lymph node metastasis (LNM) can facilitate clinical decision-making on radiotherapy or radical hysterectomy (RH) in cervical adenocarcinoma (AC)/adenosquamous carcinoma (ASC). This study aims to develop a deep learning radiomics nomogram (DLRN) to preoperatively evaluate LNM in cervical AC/ASC.

**Materials and methods:**

A total of 652 patients from a multicenter were enrolled and randomly allocated into primary, internal, and external validation cohorts. The radiomics features were extracted from axial T2-weighted imaging (T2WI), diffusion-weighted imaging (DWI), and contrast-enhanced T1-weighted imaging (CE-T1WI). The DL features from T2WI, DWI, and CE-T1WI were exported from Resnet 34, which was pretrained by 14 million natural images of the ImageNet dataset. The radscore (RS) and DL score (DLS) were independently obtained after repeatability test, Pearson correlation coefficient (PCC), minimum redundancy maximum relevance (MRMR), and least absolute shrinkage and selection operator (LASSO) algorithm performed on the radiomics and DL feature sets. The DLRN was then developed by integrating the RS, DLS, and independent clinicopathological factors for evaluating the LNM in cervical AC/ASC.

**Results:**

The nomogram of DLRN-integrated FIGO stage, menopause, RS, and DLS achieved AUCs of 0.79 (95% CI, 0.74–0.83), 0.87 (95% CI, 0.81–0.92), and 0.86 (95% CI, 0.79–0.91) in the primary, internal, and external validation cohorts. Compared with the RS, DLS, and clinical models, DLRN had a significant higher AUC for evaluating LNM (all P < 0.005).

**Conclusions:**

The nomogram of DLRN can accurately evaluate LNM in cervical AC/ASC.

## Introduction

Radical hysterectomy (RH) is widely recognized as the established standard surgical approach in early-stage cervical cancer (1). According to the 2018 version of the International Federation of Gynecology and Obstetrics (FIGO) staging system, if lymph node metastasis (LNM) is detected through preoperative imaging or pathology, the cancers will be classified as stage IIIC (2). Instead of RH, definitive radiotherapy is considered the preferred treatment approach for patients diagnosed with stage IIIC (3). On the other hand, with the routine application of RH, approximately 90% of non-metastatic lymph nodes (LN) are routinely dissected, causing various complications, such as lymphedema, neurovascular injury, and adhesion (4). Although a satisfactory 5-year survival rate is observed in early-stage cervical cancer after RH, the occurrence of LNM decreases the survival rate from 85%–90% to 50%–55% (5). Thus, the precise preoperative assessment of lymph node metastasis (LNM) holds great significance in personalized treatment plans and prognosis evaluation for patients with cervical cancer (6).

Adenocarcinoma (AC) and adenosquamous carcinoma (ASC) account for approximately 20%–25% of cervical cancers and have exhibited a rise in morbidity and mortality over the past decades, particularly in young women (6–9). Compared with squamous cell carcinoma (SCC), both AC and ASC are independent risk factors for the lower overall survival (OS) and recurrence-free survival (RFS) (7). Additionally, ASC more commonly exhibits peripheral nerve infiltration, whereas AC is linked to earlier FIGO stage, smaller tumor volume, less lymphovascular space invasion (LVSI), less deep stromal invasion (DSI), and a higher incidence of ovarian metastasis (10). The predictive factors for LNM between AC components and SCC are different (11). Therefore, it is necessary to explore reliable predictive factors and construct a powerful comprehensive model to evaluate LNM in cervical AC and ASC.

The dissected lymph nodes (LNs) can be categorized into three groups based on the size of the metastases on pathology: (1) individual tumor cells measuring less than 0.2 mm; (2) micrometastasis ranging from 0.2 mm to 2 mm; and (3) macrometastasis measuring greater than 2 mm (12). However, both the preoperative MRI and positron emission tomography (PET)-CT demonstrate a limited discriminating ability for the individual tumor cells and micrometastasis in LN with a normal size (13, 14). As parts of artificial intelligence, medical image-based radiomics and deep learning (DL) have attracted increasing attention and shown optimistic prospects in precision medicine. Radiomics is the process of the quantitative high-dimensional features extraction from medical images based on high-throughput techniques that helps to achieve precision medicine (15). Compared with radiomics, DL can achieve automatic learning and hierarchically organized task-adaptive image features and form automatic predictions for precision medicine (16).

Given the distinct biological properties and the rising morbidity and mortality of AC and ASC, the objective of this study was to construct a DL radiomics nomogram (DLRN) for the accurate evaluation of LNM in cervical AC and ASC.

## Patients and methods

The approval of this retrospective study was obtained from the institutional review board, and the informed consent requirement was waived.

The inclusion criteria were as follows: (1) cervical AC or ASC confirmed by pathology; (2) pelvic MRI examination performed within 2 weeks prior to RH or trachelectomy; (3) availability of complete clinicopathological data; (4) no concurrent malignancies. The exclusion criteria were as follows: (1) chemoradiotherapy before surgery; (2) tumor size less than 1 cm; (3) obvious imaging artifacts; (4) incomplete MRI sequences. The lymph node status was pathologically determined based on the tissue samples from the radical trachelectomy or hysterectomy.

**Figure 1:**
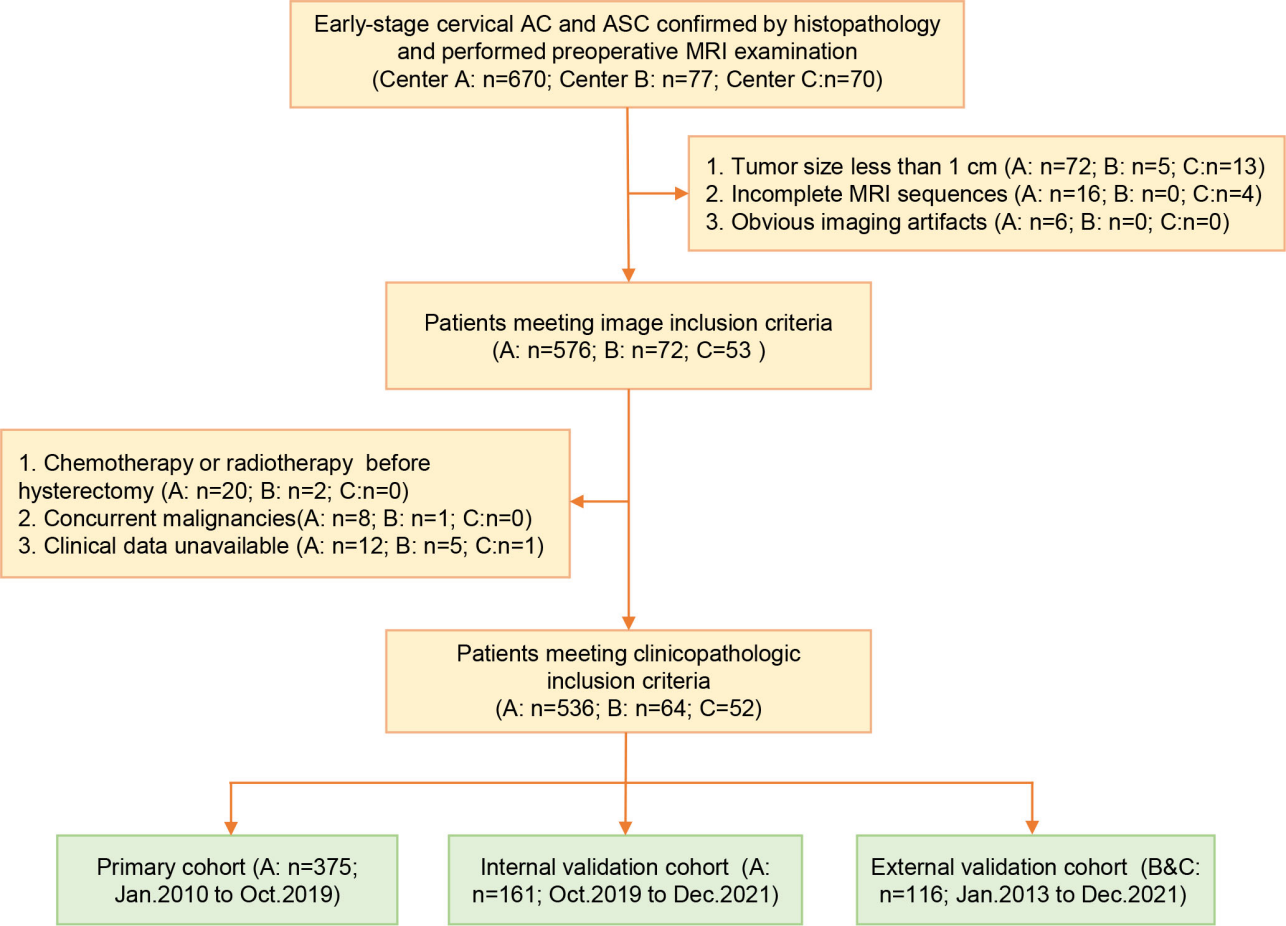
The workflow of patients’ inclusion, exclusion, and grouping.

Finally, a total of 652 patients were enrolled and allocated into the primary cohort (center A, n1 = 375, from January 2010 to October 2019), internal validation cohort (center A, n1 = 161, from October 2019 to December 2021), and external validation cohort (center B and C, n1 = 116, from January 2013 to December 2021). The patient selection process and sample size consideration are independently presented in **Figure 1** and **Supplementary 1** in **Supplementary Data Sheet 1**. The baseline data were extracted from the medical records. The tumor diameter, LNM, disruption of the cervical stromal ring (DCSR), and parametrial invasion (PMI) were measured and assessed on MRI, and the MRI definition of the above features is illustrated in **Supplementary 1** in **Supplementary Data Sheet 1**.

### MRI examination

The MRI examination was performed on 1.5 T systems (MAGNETOM Avanto, Siemens Healthineers, Erlangen, Germany; Optima 360, General Electric Healthcare, Milwaukee, WI, USA). The detailed MRI parameters are listed in **Table 1**.

**Table 1:** The detailed sequences and parameters of MR scan systems.

Parameters	Center 1			Center 2			Center 3		
	Siemens 1.5T Avanto			General Electric 1.5T Optima 360			Siemens 1.5T Avanto		
	T2WI	DWI (0,1000)	CE-T1WI	T2WI	DWI (0,800)	CE-T1WI	T2WI	DWI (50,800)	CE-T1WI
Sequence	SE	EPI	VIBE	FSE	EPI	LAVA	SE	EPI	VIBE
Repetition time (ms)	4760~5170	4000~5000	4.89	2677~2723	3700~4000	3.8~3.9	4290~4500	5100	4.49
Echo time (ms)	83	83	2.38	60.32~61.68	73~76	1.7~1.8	82	80	2.19
Field of view (mm)	280*340	200*280	280*340	280*340	200*280	280*340	280*340	200*280	280*340
Slice thickness (mm)	4~5	4~5	4~5	5~6	5~6	5~6	4~5	4~5	4~5
Gap (mm)	0.8~1	0.8~1	0.8~1	1~1.2	1~1.2	1~1.2	0.8~1	0.8~1	0.6~1

CE, contrast-enhanced; DWI, diffusion-weighted imaging; EPI, echo-planar imaging; FSE, fast spin echo; LAVA, liver acquisition with volume acceleration; SE, spin echo; T1WI, T1-weighted imaging; T2WI, T2-weighted imaging; VIBE, volumetric interpolated breath-hold examination.

### Image segmentation and preprocessing

**Figure 2:**
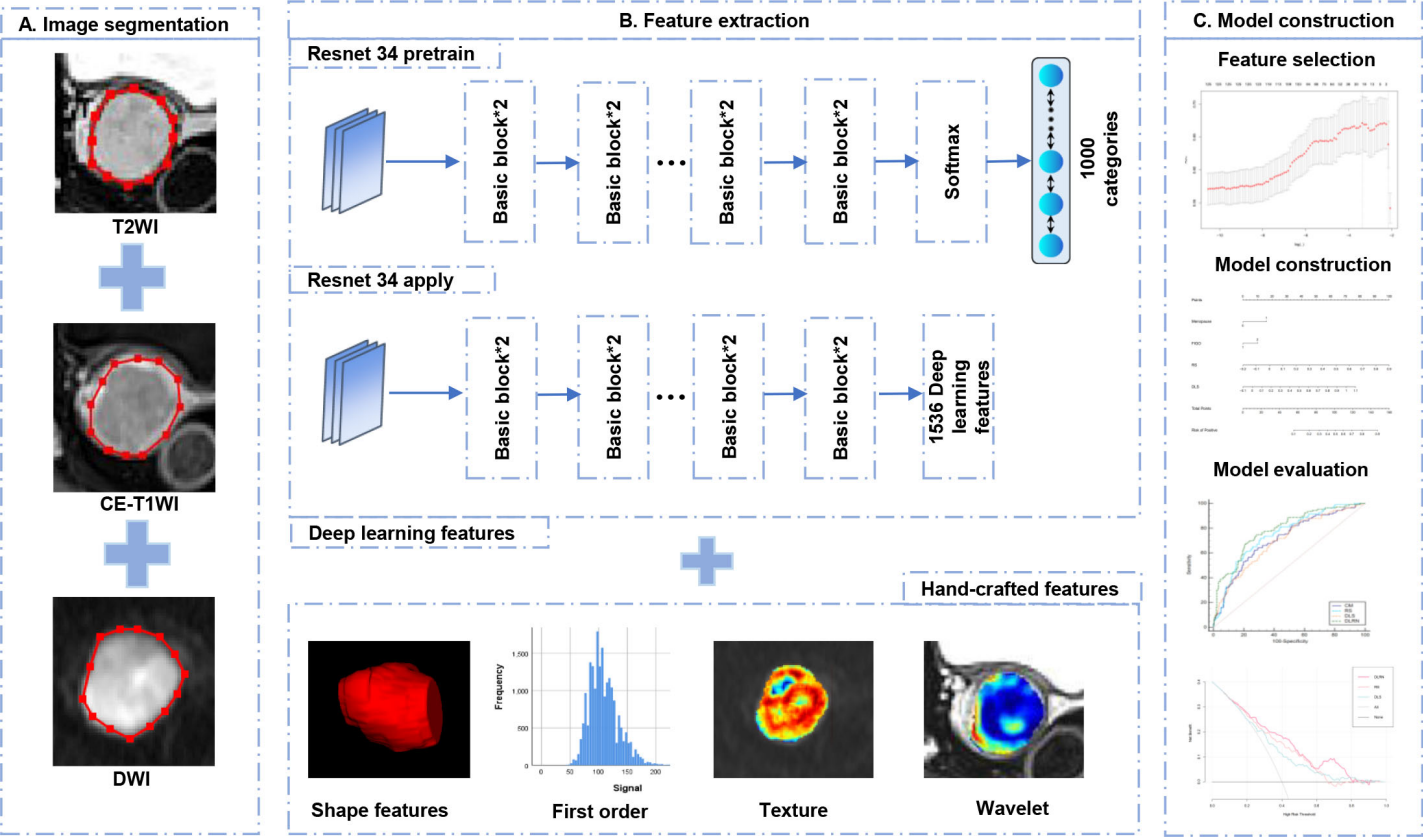
Deep learning radiomics nomogram construction and evaluation.

The regions of interest (ROIs) were manually delineated along the tumor margin on the slice of T2-weighted imaging (T2WI), diffusion-weighted imaging (DWI), and CE-T1WI with the largest tumor based on ITK-SNAP (Version 3.8.0) by Radiologist 1 (with 7 years of experience in pelvic MR imaging). Normalization was performed on T2WI and CE-T1WI by rescaling the gray values within the range of 0–800 (17). To evaluate the features repeatability, 30 patients were randomly selected and delineated by Radiologist 2 (with 5 years of experience in pelvic MR imaging) and by Radiologist 1 repeatedly 1 month later after initial segmentation. The features with intra- and inter-class correlation coefficients (ICCs) more than 0.75 were considered the reliable features and reserved for further data analysis. The construction workflow of the DLRN can be observed in **Figure 2**.

### Radiomics and deep learning features extraction and selection

Pyradiomics (version 3.0.1) was adopted to extract all radiomics features, including shape, first-order statistics, texture, and wavelet-transform features. The texture features consist of gray-level cooccurrence matrix (GLCM), gray-level run-length matrix (GLRLM), gray-level-size-zone matrix (GLSZM), gray-level-dependence matrix (GLDM), and neighborhood-gray-tone-difference matrix (NGTDM). The ResNet 34 architecture was adopted to extract DL features on PyTorch Lightning (Version 1.6.4). To make full use of the DL features, the full connection and softmax layers of the ResNet 34 were removed, and the output value of the last layer node was defined as the DL features. Then, the Z-score normalization was applied to each radiomics and DL feature by subtracting the mean and dividing by the standard error.

The following feature selection steps were performed respectively on radiomics and DL feature sets in the primary cohort: (1) Pearson correlation coefficient (PCC) of any pair of features was calculated. If PCC was greater than 0.9, one of pair features will be randomly eliminated to reduce redundancy (18). (2) The 125 highest-ranking features strongly correlated with LNM were selected by using maximum relevance and minimum redundancy (MRMR) (19). (3) The least absolute shrinkage and selection operator (LASSO) algorithm was applied with 10-fold cross-validation using the minimum criteria (1-SE criteria) to select the optimal feature set from the primary cohort. After the LASSO method, the radiomics and deep learning signature were generated, which were represented as radscore (RS) and deep learning score (DLS). The RS and DLS were independently obtained by the linear combination of selected optimal features and the corresponding weight coefficients.

### Deep learning radiomics nomogram construction and evaluation

The independent risk factors for LNM in cervical AC/ASC were identified using multivariate analysis by inputting significant variables found using univariate analysis. Backward stepwise selection was applied, in which the stopping rule was the likelihood ratio test with Akaike’s information criterion. The DLRN and clinical models were then constructed by integrating their respective independent risk factors based on logistic regression analysis. The receiver operating characteristic (ROC) curve analysis was performed on the RS, DLS, clinical model, and DLRN to assess their diagnostic abilities. The area under the curve (AUC), sensitivity (SEN), specificity (SPE), positive/negative predictive values (PPV/NPV), and positive/negative likelihood ratios (+LR/−LR) were calculated in the primary, internal, and external validation cohorts. The calibration curves, measuring how similar the evaluation outcome was to the observed ones, were drawn based on the Hosmer–Lemeshow (H–L) test. Decision curve analysis was plotted to evaluate the clinical effectiveness of DLRN by calculating the net benefits at varied threshold probabilities (20).

### Statistical analysis

R software (version 3.5.0, http://www.Rproject.org) and SPSS (version 26.0.0.0; IBM Corp., USA) were used to implement the statistical analyses. The normality was assessed based on the Kolmogorov–Smirnov test. Continuous variables with normal and non-normal distribution were expressed as mean ± standard deviation (SD) and median with interquartile range, respectively. The differences of variables between LNM positive and negative groups were assessed based on Student’s t-test, Mann–Whitney U test, or chi-squared test. The DeLong test was employed to compare the diagnostic performances of different models. A p-value of statistical significance level was set as 0.05.

## Results

### Patients

The clinicopathological characteristics of the LNM-positive and -negative patients in the primary, internal, and external validation cohorts are compared in **Table 2**. The menopause, FIGO stage, tumor diameter, and PMIMR and were determined as clinical independent risk factors and were used to build the clinical model (**Table 3**). The clinical mode achieved AUCs of 0.71 (95% CI, 0.66–0.76), 0.75 (95% CI, 0.67–0.81), and 0.71 (95% CI, 0.61–0.79) in the primary, internal, and external validation cohorts.

**Table 2:** The characteristic comparisons of LNM-positive and LNM-negative in the primary and validation cohorts.

Characteristics	Primary cohort (n=375)		P-value	Internal validation cohort (n=161)		P-value	External validation cohort (n=116)		P-value
	LNM (-) (n=269)	LNM (+) (n=106)		LNM (-) (n=111)	LNM (+) (n=50)		LNM (-) (n=88)	LNM (+) (n=28)	
Mean age (year)	46.9 ± 9.5	48.5 ± 10.6	0.161	48.5 ± 9.4	49.0 ± 10.0	0.756	49.3 ± 10.1	52.3 ± 9.9	0.174
Malignancy Family history			0.439			0.455			0.741
(+)	19 (7.1%)	10 (9.4%)		13 (11.7%)	8 (16.0%)		10 (11.4%)	4 (14.3%)	
(–)	250 (92.9%)	96 (90.6%)		98 (88.3%)	42 (84.0%)		78 (88.6%)	24 (85.7%)	
Fertility			0.303			0.518			0.675
(+)	256 (95.2%)	98 (92.5%)		104 (93.7%)	45 (90.0%)		83 (94.3%)	26 (92.9%)	
(–)	13 (4.8%)	8 (7.5%)		7 (6.3%)	5 (10.0%)		5 (5.7%)	2 (7.1%)	
Menopause			0.011			0.312			0.106
(+)	82 (30.5%)	47 (44.3%)		46 (41.4%)	25 (50.0%)		38 (43.2%)	17 (60.7%)	
(–)	187 (69.5%)	59 (55.7%)		65 (58.6%)	25 (50.0%)		50 (56.8%)	11 (39.3%)	
Histology			0.288			0.818			0.536
AC	141 (52.4%)	62 (58.5%)		51 (45.9%)	22 (44.0%)		65 (73.9%)	19 (67.9%)	
ASC	128 (47.6%)	44 (42.5%)		60 (54.1%)	28 (56.0%)		23 (26.1%)	9 (32.1%)	
FIGO stage			0.000			0.018			0.969
IB1-IB2	219 (81.4%)	62 (58.5%)		100 (90.1%)	38 (76.0%)		72 (81.2%)	23 (82.1%)	
IIA-IIB	50 (18.6%)	44 (42.5%)		11 (9.9%)	12 (24.0%)		16 (18.8%)	5 (17.9%)	
Tumor diameter on MRI (cm)	3.35 ± 1.21	3.96 ± 1.12	0.000	3.31 ± 1.15	4.26 ± 1.12	0.000	3.32 ± 1.11	4.01 ± 1.14	0.008
PMIMR			0.000						0.000
(+)	32 (11.9%)	36 (34.0%)		12 (10.8%)	21 (42.0%)	0.000	11 (12.5%)	12 (42.9%)	
(–)	237 (88.1%)	70 (66.0%)		99 (89.2%)	29 (58.0%)		77 (87.5%)	16 (57.1%)	
DCSRMR			0.004			0.000			0.334
(+)	133 (49.4%)	70 (66.0%)		48 (43.2%)	40 (80.0%)		54 (61.4%)	20 (71.4%)	
(–)	136 (50.6%)	36 (34.0%)		63 (56.8%)	10 (20.0%)		34 (38.6%)	8 (28.6%)	
LNMMR			0.001			0.000			0.199
(+)	82 (30.5%)	52 (49.1%)		19 (17.1%)	25 (50.0%)		32 (36.4%)	14 (50.0%)	
(–)	187 (69.5%)	54 50.9(%)		92 (82.9%)	25 (50.0%)		56 (63.6%)	14 (50.0%)	
PMI			0.000			0.000			0.000
(+)	16 (5.9%)	32 (30.2%)		1 (0.1%)	14 (28.0%)		3 (3.4%)	9 (32.1%)	
(–)	253 (94.1%)	74 (69.8%)		110 (99.9%)	36 (72.0%)		85 (96.6%)	19 (67.9%)	
DSI			0.000			0.000			0.000
(+)	177 (65.8%)	92 (84.9%)		56 (50.5%)	4 (8%)		35 (39.8%)	23 (82.1%)	
(–)	92 (34.2%)	14 (13.2%)		55 (49.5%)	46 (92.0%)		53 (60.2%)	5 (17.9%)	
DLS	0.250.13	0.36 ± 0.17	0.000	0.24 ± 0.18	0.47 ± 0.24	0.000	0.19 ± 0.16	0.41 ± 0.22	0.000
RS	0.24 ± 0.16	0.40 ± 0.15	0.000	0.23 ± 0.18	0.50 ± 0.18	0.000	0.19 ± 0.16	0.38 ± 0.19	0.000

AC, adenocarcinoma; ASC, adenosquamous carcinoma; DSI, deep stromal invasion; DCSRMR, disruption of cervical stroma ring on MRI; DLRS, deep learning radiomics score; DLS, deep learning score; FIGO, International Federation of Gynecology and Obstetrics (2009); LNM, lymph node metastasis. LNMMR, lymph node metastasis on MRI; PMI, parametrial invasion; PMIMR, parametrial invasion on MRI; RS, radscore.

**Table 3:** The construction of the clinical model and nomogram using multivariate logistic regression analysis.

Variables	Clinical model			Nomogram		
	β	Odds Ratio	P-value	β	Odds Ratio	P-value
Menopause	0.58	1.79 (1.08-2.97)	0.025	0.07	2.16 (1.27-3.70)	0.005
FIGO stage	0.68	1.98 (1.16-3.40)	0.013	0.48	1.61 (0.92-2.82)	0.093
Tumor diameter	0.29	1.33 (1.07-1.65)	0.012	/	/	/
PMIMR	0.72	2.05 (1.10-3.85)	0.025	0.18	1.19 (0.60-2.39)	0.619
DCSRMR	-0.06	0.94 (0.54-1.63)	0.833	-0.3	0.74 (0.42-1.32)	0.312
LNMMR	0.38	1.47 (0.87-2.48)	0.155	0.23	1.25 (0.72-2.19)	0.428
RS	/	/	/	4.36	78.25 (12.48-490.59)	0.000
DLS	/	/	/	3.08	21.72 (3.39-139.13)	0.001
Consistant	-3.23	0.04	< 0.000	-4.16	0.02	0.000

### Feature selection

**Figure 3:**
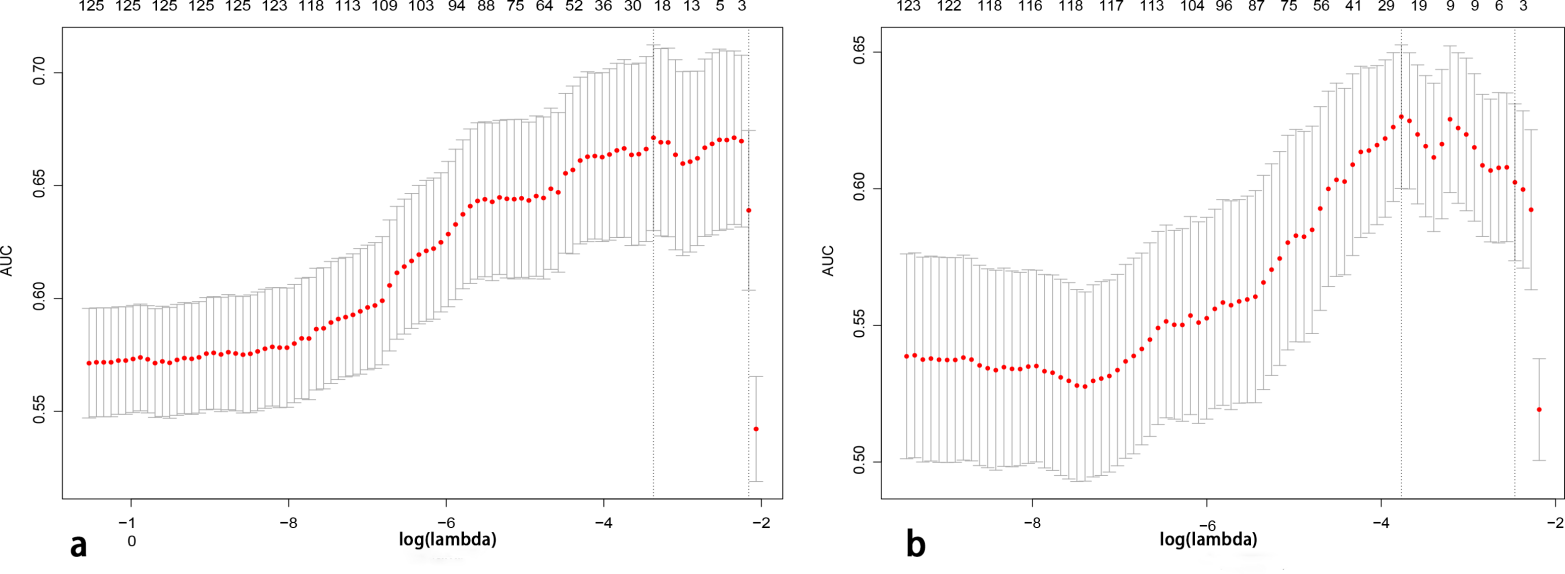
Features determined by the LASSO algorithm. The lambda (λ) is selected based on 10-fold cross-validation via minimum criteria (1-SE criteria). **(A, B)** A total of 22 and 25 features were selected from radiomics and deep learning feature sets (LASSO, least absolute shrinkage and selection operator).

**Figure 4:**
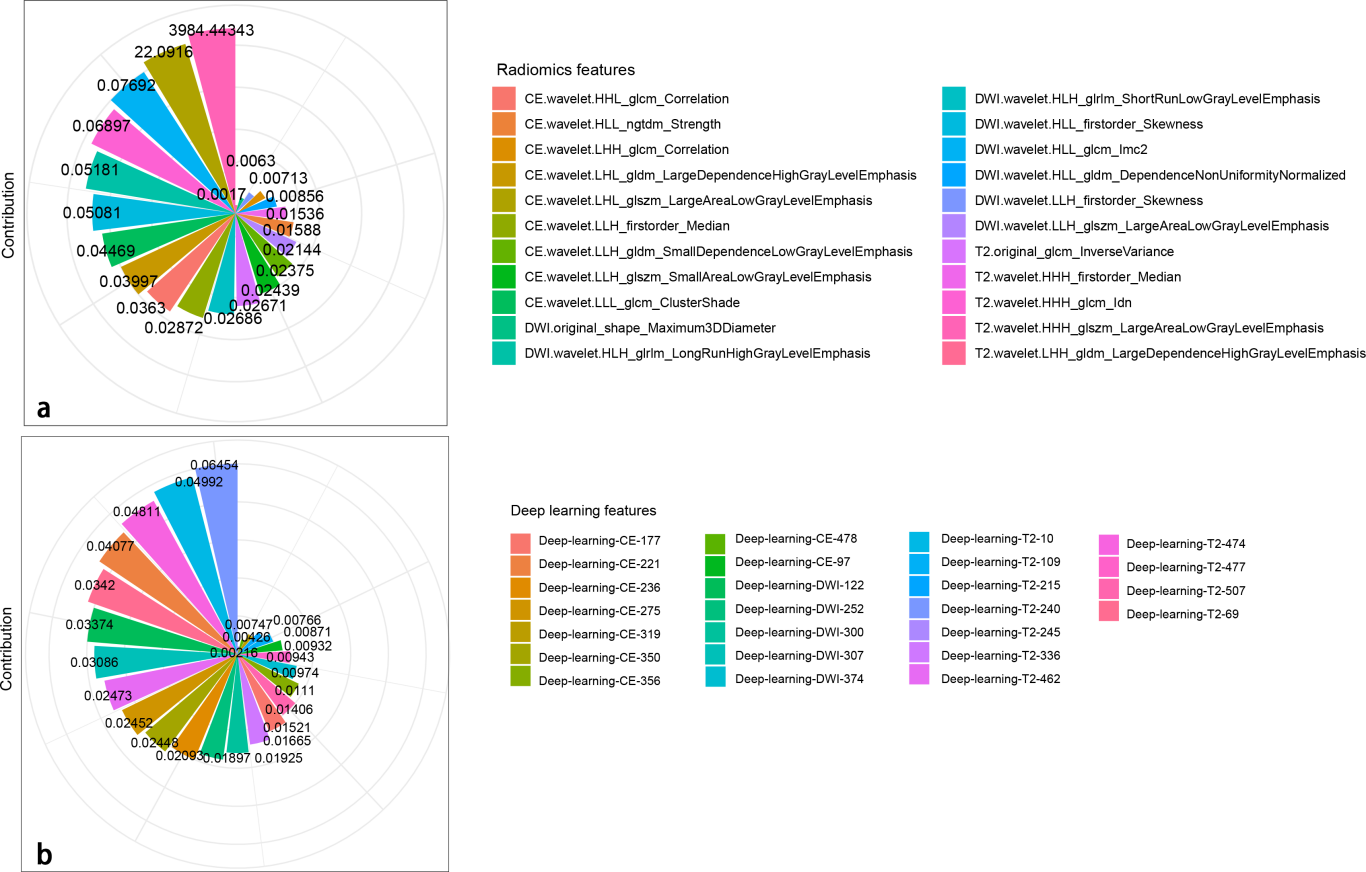
Radiomics **(A)** and DL **(B)** features with their respective weight coefficients after LASSO regression.

**Figure 5:**
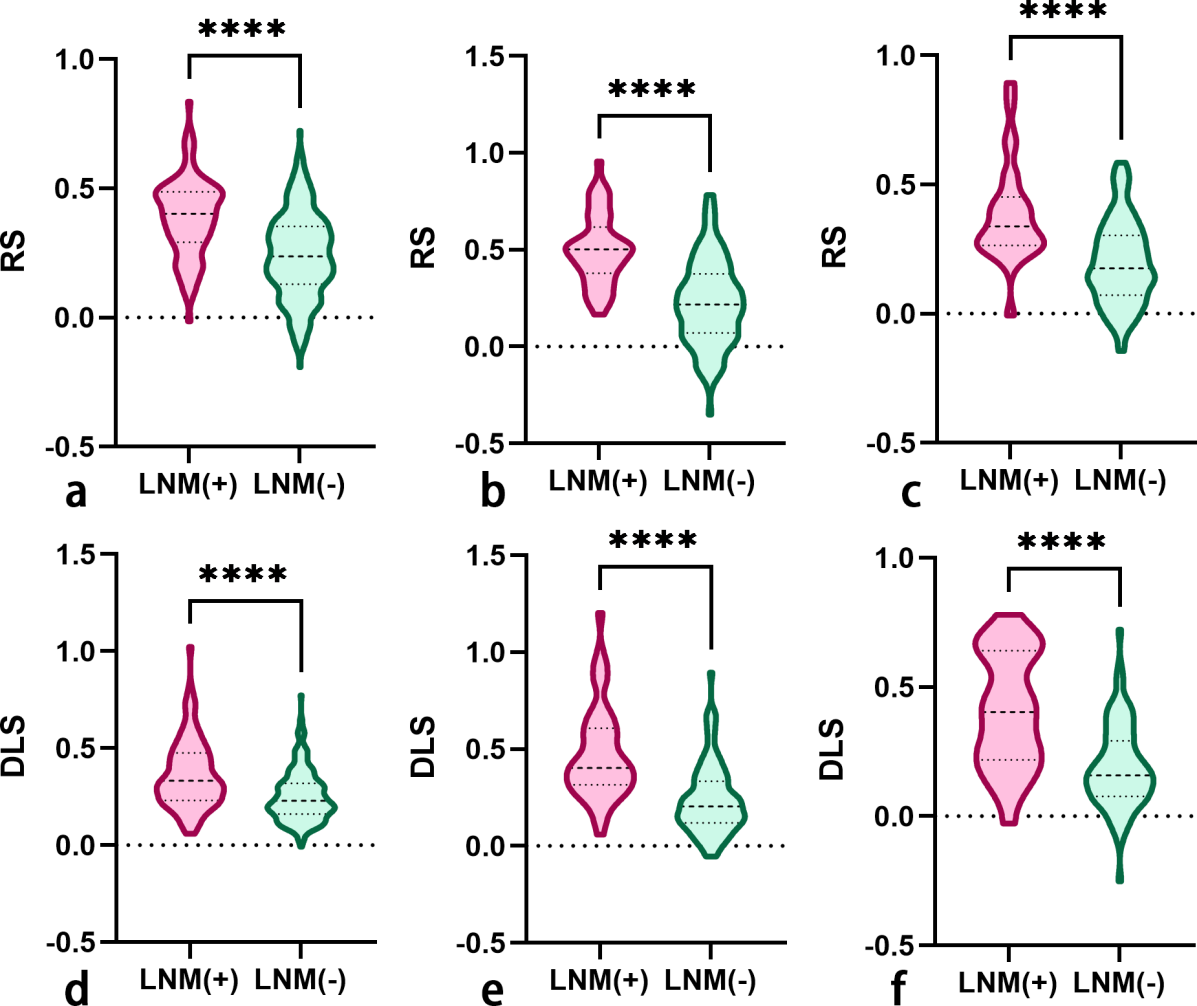
The RS **(A–C)** and DLS **(D–F)** differences between LNM-positive and LNM-negative groups in the primary, internal validation, and external validation cohorts (RS, radscore; DLS, deep learning score; LNM, lymph node metastasis). The symbol **** means P-value less than 0.0001.

A total of 2,499 radiomics features were extracted from ROIs of T2WI, DWI, and CE-T1WI. After ICC, PCC, MRMR, and LASSO algorithm, the 22 most optimal features were reserved. A total of 1,536 DL features were extracted from ROIs of T2WI, DWI, and CE-T1WI. After ICC, PCC, MRMR, and LASSO algorithms, the 25 most optimal features were reserved. **Figures 3**, **4** show the feature selection process, optimal radiomics and DL feature sets and their respective weight coefficients. After linear combination with their respective weight coefficients, the RS and DLS were independently calculated. The RS and DLS differences between LNM-positive and LNM-negative groups in three cohorts are shown in **Figure 5**.

### The evaluation performance and comparison of RS and DLS

For evaluating LNM, the AUCs of RS and DLS were 0.75 (95% CI, 0.70–0.79) and 0.70 (95% CI, 0.65–0.75) in the primary cohort, were 0.84 (95% CI, 0.77–0.89) and 0.81 (95% CI, 0.74-0.96) in the internal validation cohort, and were 0.79 (95% CI, 0.70–0.86) and 0.78 (95% CI, 0.69–0.85) in the external validation cohort, respectively. The DeLong test showed that there was a comparable performance for evaluating LNM between RS and DLS in the primary (P = 0.096), internal validation (P = 0.452), and external validation cohorts (P = 0.936). Therefore, both the RS and DLS were selected to construct the DLRN.

### The construction and evaluation of DLRN

**Figure 6:**
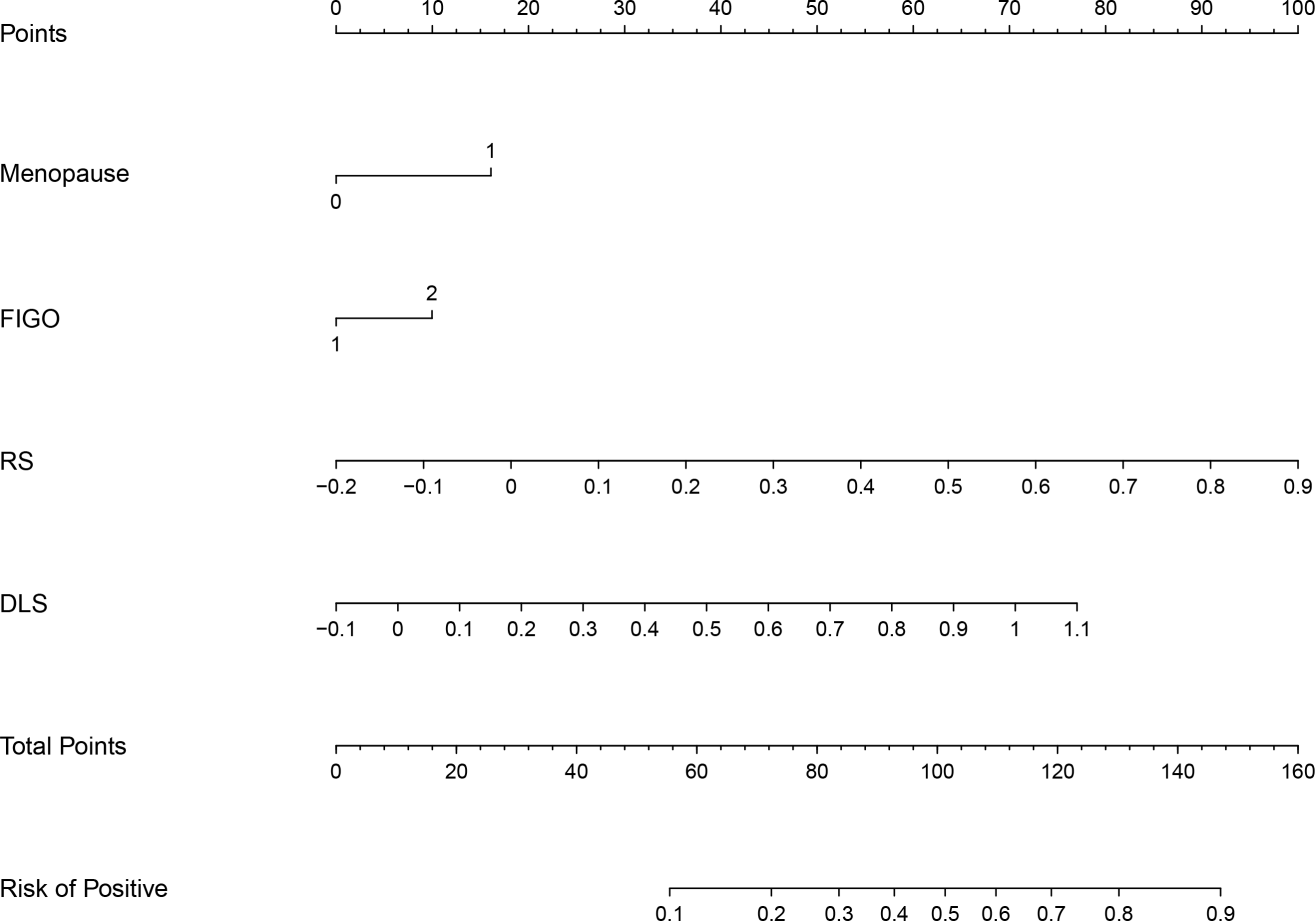
The DLRN based on RS, DLS, and independent risk factors of menopause and FIGO stage (FIGO, International Federation of Gynecology and Obstetrics).

**Figure 7:**
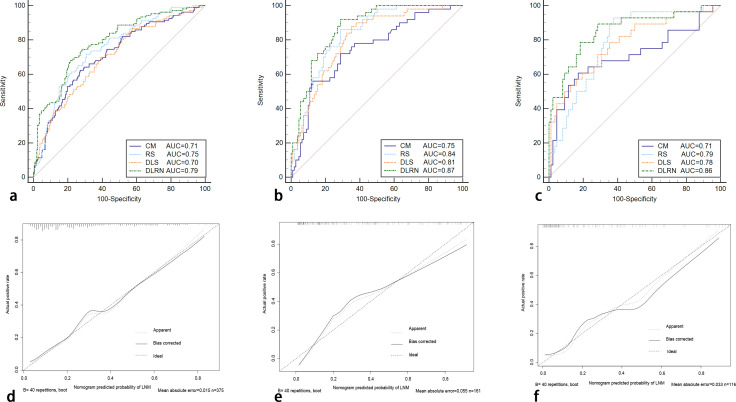
The ROC curves of RS, DLS, clinical model, and DLRN for predicting LNM in the primary **(A)**, internal **(B)**, external validation **(C)** cohorts. The calibration curves of the DLRN in the primary cohort [**(D)**, P = 0.913], internal validation [**(E)**, P = 0.958], and external validation cohorts [**(F)**, P = 0.803] ROC, receiver operator characteristic; CM, clinical model.

In the process of constructing the nomogram, the RS, DLS, menopause, and FIGO stage were determined as the independent risk factors. The DLRN was constructed by integrating RS, DLS, menopause, and FIGO stage (**Figure 6**). The AUC, SEN, and SPE of DLRN were 0.79% (95% CI, 0.74–0.83), 67.9%, and 78.4% in the primary cohort; 0.87% (95% CI, 0.81–0.92), 92.0%, and 71.2% in the internal validation cohort; 0.86% (95% CI, 0.78–0.91), 89.3%, and 71.6% in the external validation cohort, respectively. The AUC of DLRN was significantly higher than those of RS, DLS, and clinical model in the primary and internal validation cohorts (all P < 0.05) than that of the clinical model in the external validation cohort (P = 0.011). The comparison details are presented in **Figures 7A–C** and **Table 4**. The H-L tests demonstrated that evaluation outcomes of the DLRN had an ideal agreement with actual observation ones in the primary (P=0.913), internal validation (P=0.958), and external validation cohorts (P = 0.803). The calibration curves of DLRN are shown in **Figures 7D–F**.

**Table 4:** The diagnostic performance of DLRN, DLS, RS, and clinical models for predicting the LNM in patients with cervical AC and ASC.

Cohorts	Model	AUC	P-value	SEN	SPE	+LR	-LR	PPV	NPV
Primary	DLRN	0.79 (0.74-0.83)	/	67.9	78.4	3.2	0.4	55.4	86.1
	DLS	0.70 (0.65-0.75)	0.000	69.8	61.0	1.8	0.5	41.3	83.7
	RS	0.75 (0.70-0.79)	0.023	71.7	69.1	2.3	0.4	47.8	86.1
	Clinical	0.71 (0.66-0.76)	< 0.000	62.3	72.9	2.3	0.5	47.5	83.1
Internal validation	DLRN	0.87 (0.81-0.92)	/	92.0	71.2	3.2	0.1	59.0	95.2
	DLS	0.81 (0.74-0.96)	0.022	88.0	64.9	2.5	0.2	53.0	92.3
	RS	0.84 (0.77-0.89)	0.037	86.0	71.2	3.0	0.2	57.3	91.9
	Clinical	0.75 (0.67-0.81)	0.001	56.0	87.4	4.4	0.5	66.7	81.5
External validation	DLRN	0.86 (0.78-0.91)	/	89.3	71.6	3.1	0.2	50.0	95.5
	DLS	0.78 (0.69-0.85)	0.067	78.6	64.8	2.2	0.3	41.5	90.5
	RS	0.79 (0.70-0.86)	0.068	92.9	62.5	2.5	0.1	44.1	96.5
	Clinical	0.71 (0.61-0.79)	0.011	60.7	83.0	3.6	0.5	53.1	86.9

DLRN, deep learning radiomics nomogram; AUC, the area under the curve; +LR, positive likelihood ratio; –LR, negative likelihood ratio; NPV, negative predictive value; PPV, positive predictive value; SEN, sensitivity; SPE, specificity.

### Clinical application of nomogram

**Figure 8:**
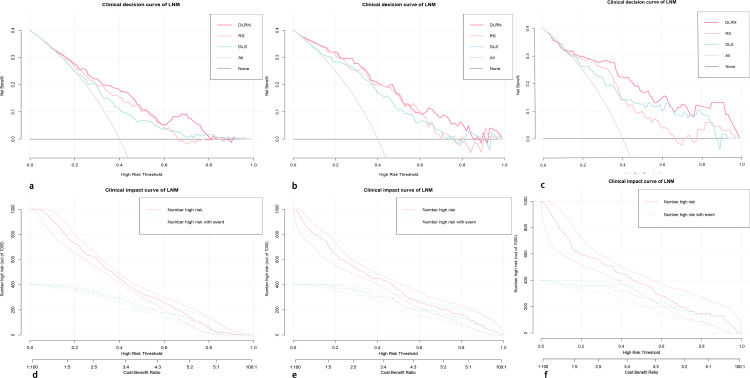
The clinical decision curves **(A–C)** and clinical impact curves **(D–F)** of the DLRN in the primary, internal, and external validation cohorts. In the external validation cohort, when the threshold probability is within the range of 0.12–0.98, the use of DLRN can obtain more net benefits for patients than the “all” or “none” treatment scheme.

The clinical decision curve along with clinical impact curve of DLRN in the primary internal validation and external validation cohorts are shown in **Figure 8**. In the external validation cohort, when the threshold probability was within the range of 0.12–0.98, the use of DLRN added more net benefits than the scheme of “treatment-all” or “treatment-none”. In the most range of the threshold probability, the net benefit of DLRN was more than those of DLS, RS, and clinical model.

## Discussion

In this study, the DLRN, by integrating the DLS, RS, and clinical independent risk factors, could be helpful for assessing the LNM in patients with AC/ASC. The AUCs of DLRN were 0.79 (95% CI, 0.74–0.83), 0.87 (95% CI, 0.81–0.92) and 0.86 (95% CI, 0.78–0.91) in the primary, internal and external validation cohorts, respectively.

The conventional MRI morphological criteria for the diagnosis of LNM are as follows: short axis diameter > 8 mm, round shape, irregular contour, lack of fat hilus on T2WI, hyperintensity on DWI and heterogeneous enhancement on CE-T1WI (18). The conventional MRI morphological criteria had a combined SEN of 51% and SPE of 90% for evaluating the LNM in cervical cancer (13). The radiomics nomogram could accurately diagnose the LNM in cervical SCC, with the C index ranging from 0.856 to 0.909 (3, 21). A previous cervical AC/ASC study showed that the AUC of the support vector machine model for evaluating LNM was 0.79 and 0.82 in the primary and validation cohorts, respectively (19). This study had the following differences compared with aforementioned studies: (1) the sufficient multicenter samples; (2) focusing on cervical AC/ASC; (3) the established DLRN containing multiscale information (radiomics features, DL features, and clinical characteristics).

The radiomics features highly depend on the accurate tumor segmentation. The features extracted from different manual segmentations of the same lesion may vary widely (22). Compared with radiomics, DL can achieve automatic learning and hierarchically organized task-adaptive image features. To select reliable features with good reproducibility, both the radiomics and DL features with ICCs less than 0.75 were eliminated. Meanwhile, considering the possibility of redundancy between radiomics and DL features, the PCC test was performed to remove redundant features.

In the diagnostic performance comparison of radiomics features and DL features, consistent conclusion has not been reached yet. Some studies concluded that the diagnostic performance of DL features was inferior to that of the radiomics features (23, 24), whereas another study achieved a different conclusion (25). In this study, the DLS yielded a comparable diagnostic performance to the RS with no significant difference between two signatures. Therefore, both RS and DLS were adopted to construct the comprehensive nomogram.

Previous studies demonstrated that the FIGO stage and tumor size were clinical independent risk factors and were integrated into the model to evaluate LNM in cervical SCC (3, 21, 26). In this study, the factors of menopause, FIGO stage, tumor diameter, and PMIMR were determined as clinical independent risk factors and were used to build the clinical model. In the process of constructing a comprehensive nomogram, tumor diameter was excluded because of the same information that had been contained in the optimal radiomics signature. Finally, menopause, FIGO stage, RS, and DLS were determined as independent risk factors and were used to build the DLRN.

The overfitting issue needed to be carefully dealt with in the process of model building. In this study, the risk of overfitting of DLRN was low. The reasons were as follows: (1) the AUCs of DLRN were greater in the validation cohorts than in the primary cohort, which was a reliable evidence for eliminating overfitting bias. (2) To reduce the risk of overfitting, we had adopted the following guidelines in our logistic regression model: the predictor number should generally be within 1/20 to 1/8 of sample sizes of the training cohort, and the event-per-predictor ratio should remain within the range of 5 to 9 (27, 28).

The potential reasons that the accuracy of primary cohort was lower than validation cohorts are as follows: (1) To avoid the influence of random effects on model construction, the non-random method based on time grouping was adopted to construct the DLRN, which was widely used in authoritative research articles (29–31). However, this time grouping-based non-random approach led to an imbalanced distribution of features across the cohorts. (2) The morbidity of the AC/ASC was significantly lower than that of SCC. The large number of AC/ASC cases needs to collect from multiple centers over an extended period. In this study, a total of 536 patients from center A were enrolled and allocated into the primary cohort (from January 2010 to October 2019) and internal validation cohort (from October 2019 to December 2021). The data selection bias among primary and validation cohorts that from different time were inevitable due to its retrospective nature. Therefore, the imbalanced distribution of optimal clinical, radiomic, and deep learning features selected among the primary and validation cohorts was the primary factor contributing to the lower accuracy in the training data.

The nomogram is a visual tool used to generate personalized probability predictions. Gynecologists and radiologists can make preoperative individualized predictions of the risk of LNM using this user-friendly scoring system, aligning with the current trend toward personalized precision medicine. In this study, the RS, DLS, FIGO stage, and menopause were identified as independent risk factors and were utilized to develop the DLRN. When using the nomogram, the specific points of individual patients are plotted on each risk factor axis. A vertical line is drawn upward to determine the points assigned to each risk factor. Then, the total of these points is marked on the total points axis to indicate the probability of LNM in each patient.

This study had the following limitations. First, the data selection bias and data sampling bias were inevitable to some extent due to its retrospective nature. Furthermore, the DLRN should be validated in a prospective research with larger external sample size. Second, the serum tumor biomarkers were not used to develop a model due to incomplete data. Third, the DLRN built could not accurately evaluate the specific location of metastatic lymph nodes. Finally, instead of specific features of LN, such as edge, length, and diameter, the MRI morphological criteria for the diagnosis of LNM (LNMMR) were adopted to explore its potential value (28).

In conclusion, the DLRN, by integrating menopause, FIGO stage, RS, and DLS, achieved a significantly better performance than DLS, RS, and clinical model in evaluating LNM in patients with cervical AC and ASC. The prediction outcome derived from DLRN may facilitate clinical decision-making of radiotherapy or RH.

## Data Availability

The raw data supporting the conclusions of this article will be made available by the authors, without undue reservation.
