# Supplementary material for: The deep learning radiomics nomogram helps to evaluate the lymph node status in cervical adenocarcinoma/adenosquamous carcinoma
Source: Front Oncol. 2024 Dec 13;14:1414609. doi: 10.3389/fonc.2024.1414609 (PMC11671353; doi:10.3389/fonc.2024.1414609)
Supplement: Supplementary file 1 [file DataSheet1.docx]

**Supplement 1: The sample size calculation of the validation cohorts**

The sample sizes of lymph node metastasis (LNM)-negative and LNM-positive needed in the internal and external validation cohorts were calculated. The null hypothesis of an area under the curve (AUC) of 0.65 and an alternative hypothesis of AUC of 0.80 were proposed. The ratio of LNM-negative and LNM-positive was 2:1. Then, the smallest samples of 114 patients were needed based on a type I error of 5% with 80% power. The sample size calculation was conducted on MedCalc (Version 19.6.4, MedCalc Software Ltd).

**Supplement 2:** **The magnetic resonance imaging (MRI) definition of lymph node metastasis (LNMMR), parametrial invasion (PMIMR), and disruption of the cervical stromal ring (DCSRMR).**

**Definition of LNMMR:** The short axis of the LN measures over 8 mm, displaying a rounded shape, irregular contour, absence of a fat hilum, hyperintensity on diffusion-weighted imaging (DWI), and heterogeneous enhancement on contrast-enhanced T1-weighted imaging (CE-T1WI).^1^

**Definition of PMIMR:** PMI diagnosis necessitated both full-thickness cervical stromal infiltration and at least one supplementary characteristic on T2-weighted imaging (T2WI), such as a spiculated tumor-to-parametrial interface, presence of a tumor nodule in the parametrium, or encasement of parametrial vessels by the tumor.^2,3^

**Definition of DCSRMR:** Disruption of the outer rim of hypointense cervical stroma ring on T2WI.

The Radiologists 1 and 2 (*** and **, with 7 and 10 years of experience in gynecological imaging, respectively), who blinded to all clinical data except for the diagnosis of cervical cancer, independently assessed the tumor volume, LNMMR, PMIMR and DCSRMR by reviewing all MRI data. To calculate the reproducibility, the MRI data of all cases were re-assessed by Radiologist 1 after one month. The inter-/intra-class correlation coefficients of above MRI characteristics ranged from 0.71 to 0.78.

**Reference**

1. Xiao ML, Wei Y, Zhang J, Jian JM, Song Y, Lin ZJ, et al. MRI texture analysis for preoperative prediction of lymph node metastasis in patients with nonsquamous cell cervical carcinoma. Acad Radiol. 2022: S1076-6332(22)00021-6.

2. Sala E, Rockall AG, Freeman SJ, Mitchell DG, Reinhold C. The added role of MR imaging in treatment stratification of patients with gynecologic malignancies: what the radiologist needs to know. Radiology. 2013; 266:717-40.

3. Raithatha A, Papadopoulou I, Stewart V, Barwick TD, Rockall AG, Bharwani N. Cervical cancer staging: a resident's primer: women's imaging. Radiographics. 2016; 36:933-4.
